# Role-playing is an effective instructional strategy for genetic counseling training: an investigation and comparative study

**DOI:** 10.1186/s12909-016-0756-4

**Published:** 2016-09-02

**Authors:** Xiao-feng Xu, Yan Wang, Yan-yan Wang, Ming Song, Wen-gang Xiao, Yun Bai

**Affiliations:** 1 Department of Medical Genetics, Third Military Medical University, Chongqing, 400038 People’s Republic of China; 2 Department of Neurology, Second Affiliated Hospital, Chongqing Medical University, Chongqing, 400010 People’s Republic of China; 3 College of Basic Medical Sciences, Third Military Medical University, Chongqing, 400038 People’s Republic of China

**Keywords:** Educational and training program, Genetic counseling, Role-playing, Medical curricula

## Abstract

**Background:**

Genetic diseases represent a significant public health challenge in China that will need to be addressed by a correspondingly large number of professional genetic counselors. However, neither an official training program for genetic counseling, nor formal board certification, was available in China before 2015. In 2009, a genetic counseling training program based on role-playing was implemented as a pilot study at the Third Military Medical University to train third-year medical students.

**Methods:**

Questionnaires on participant attitudes to the program and role-playing were randomly administered to 324 students after they had finished their training. Pre- and post-training instructional tests, focusing on 42 key components of genetic counseling, were administered randomly to 200 participants to assess mastery of each component. Finally, scores in final examinations of 578 participants from 2009 to 2011 were compared to scores obtained by 614 non-participating students from 2006 to 2008 to further assess program efficacy.

**Results:**

Both the training program and the instructional strategy of role-playing were accepted by most participants. Students believed that role-playing improved their practice of genetic counseling and medical genetics, enhanced their communication skills, and would likely contribute to future professional performance. The average understanding of 40 of the key points in genetic counseling was significantly improved, and most students approached excellent levels of mastery. Scores in final examinations and the percentages of students scoring above 90 were also significantly elevated.

**Conclusions:**

Role-playing is a feasible and effective instructional strategy for training genetic counselors in China as well as in other developing countries.

**Electronic supplementary material:**

The online version of this article (doi:10.1186/s12909-016-0756-4) contains supplementary material, which is available to authorized users.

## Background

The incidence of genetic disease is relatively high in mainland China. As recently as 1990, it was estimated that 20–25 % of Chinese people had at least one genetic disorder, including 3–5 % with monogenic diseases, 15–20 % with complex diseases and 0.5–1 % with chromosomal diseases [1]. Although monogenic and chromosomal diseases are now less common, public health issues related to genetic disease remain. Because China is a developing country, environmental pollution represents an additional burden on the genetic load of the population, in which the average person may carry 5 to 6 harmful genetic mutations [1]. Genetic counseling is clearly an important strategy for dealing with these challenges in low- to middle-income countries [2, 3], and professional genetic counselors are urgently needed in China for this reason.

Prior to 2015, official educational and training programs for genetic counseling were unavailable, and board certification for genetic counselors was nonexistent [4]. Although patients or counselees can usually obtain some guidance on genetic diseases within departments of obstetrics or pediatrics [5], thousands of hospitals have no genetics clinics because there are so few trained genetic counselors in China. Therefore, medical schools have an urgent need to train undergraduates to master the professional knowledge and skills of genetic counseling.

While genetic counseling is not an unknown concept among medical genetics teachers in China, there is little experience in teaching genetic counseling training. The limited literature available suggests that role-playing may be an effective teaching method not only for physicians and genetic counselors but also undergraduates [3, 6–12]. Role-playing is widely used in medical education in areas such as procedural skills [13], communication training [14], decision making [15], active learning [16], problem-solving [17], developing empathy [18], and teamwork [19], and appears even in first-year medical courses [20, 21]. Moreover, role-playing is a low cost approach that is relatively easy to implement [22]. In order to explore teaching methods appropriate for use in China, a preliminary genetic counseling training program was integrated into the medical curriculum for undergraduates at the Third Military Medical University (TMMU) in Chongqing, China. Role-playing was used as a practical instructional method and its effectiveness was evaluated.

## Methods

### Participants

A total of 2326 medical undergraduates have participated in the training program since 2009. All participants had passed the National College Entrance Examination and were enrolled to study clinical medicine for 5 years at TMMU. The program requires 1 year of natural and social sciences, 1.5 years of basic medical sciences, 1.5 years of clinical medical sciences, and the final year is spent practicing in hospitals (Table 1). The genetic counseling training program was included in the course on Medical Genetics in the third year (Table 1). Before undertaking the face-to-face role-playing activities in genetic counseling, students had already acquired basic information concerning monogenic, complex, chromosomal and mitochondrial diseases, population and clinical genetics, and the related research methods.

**Table 1:** Core Curriculum for Medical Undergraduates at TMMU

Stages	Total study time	Main subjects	Aims
Natural and social sciences	1 year	Medical History, Medical Mathematics, Medical Physics, Medical Chemistry, English, Computer, Medical Statistics, Literature Index	To know natural and social sciences and to have basic knowledge of humanities
Basic medical sciences	1.5 years	Human Anatomy, Histology and Embryology, Physiology, Biochemistry, Molecular Biology, Medical Microbiology, Medical Immunology, Human Parasitology, Pathophysiology, Pathology, Pharmacology, **Medical Genetics**	To master the basic medical foundations of theory and to develop a basic ability to think analytically to solve clinical problems
Clinical medical sciences	1.5 years	Image Medicine, Diagnosis, Internal Medicine, Surgery, Gynecology and Obstetrics, Pediatrics, Neurology, Psychiatry, Dermatology, Ophthalmology, Otorhinolaryngology, Medical Psychology	To master the basic clinical foundations of theory and to develop a strong ability to think analytically to solve clinical problems
Clinical probation and practice	1 year	Clinical probation, Clinical general practice	To have the ability to collect medical history, communicate effectively with patients, and the skills to diagnose and treat common diseases

Bold: to emphasize Medical Genetics

### Tutors

**Fig. 1:**
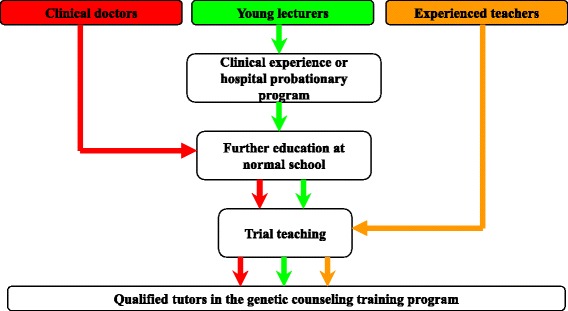
Backgrounds and supplementary preparation for tutors in the genetic counseling training program at TMMU. Clinical doctors (*red*), young lecturers (*green*) and experienced teachers (*orange*) were encouraged to participate in the genetic counseling training program at TMMU as tutors. To be well prepared, all were required to complete additional training. Young lecturers were exposed to clinical work to accumulate relevant experience. Both young lecturers and clinical doctors were required to study the knowledge and skills of pedagogy for 3–4 weeks at normal schools. All tutors, including the experienced teachers, participated in at least one trial teaching session to familiarize them with role-playing in the genetic counseling training program

This training program recruited more than 20 tutors, which included young lecturers, clinical doctors and experienced teachers. Additional training was required to be qualified as a tutor; briefly, young lecturers were required to practice clinical work at the beginning of their tenure. Both young lecturers and clinical doctors trained in normal schools for 3 to 4 weeks to improve their teaching abilities. All tutors, including the experienced teachers, were required to satisfactorily complete a trial teaching exercise (Fig. 1).

### Educational design

The goal of the program was designed to help undergraduates to apply the learned knowledge to deal with the clinical problems, to develop communication skills by play their roles, and to raise their interest in genetics. The integral program had three stages, including *i*) studying the theoretical knowledge of medical genetics, *ii*) preparing the case scenarios, and *iii*) playing the roles in the classrooms.

A pre-training instructional test was first administered to the randomly selected participants. After studying the 30 h basic theories of medical genetics by traditional lectures and problem-based learning, learning groups were self-organized by 2 or more undergraduates, and each group was a unit within the program.

Three distinct case scenarios were selected for role-playing, presenting three basic clinical situations:

i) Phenylketonuria: a new couple is very anxious that their unborn child might have phenylketonuria since the bride’s brother has the disorder.
ii) Hemophilia A: a counselee and his two brothers share symptoms including frequent bleeding without normal blood clotting, and swollen and painful knees that make walking difficult. The counselee and his caregiver want to know whether these problems would be inherited.
iii) β-thalassemia: a couple is afraid of giving birth to a child with β-thalassemia and want to know if a genetic diagnosis would help them.

**Fig. 2:**
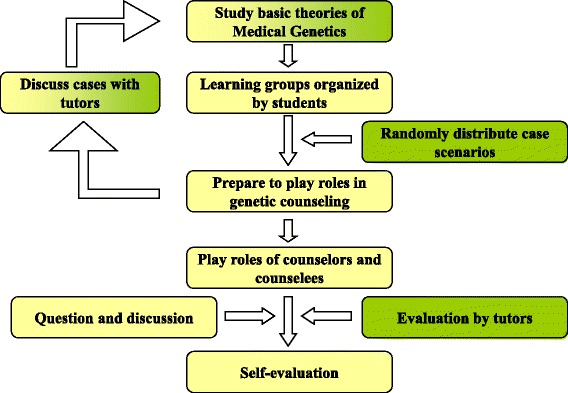
Overview of the genetic counseling training program at TMMU. Third-year medical undergraduates participated in a student-centered genetic counseling training program at TMMU. Before participating face-to-face in role-playing, they had already studied the fundamentals of medical genetics. One of three case scenarios for role-playing was randomly assigned to a learning group which had been organized by two or more undergraduates. The learning groups prepared the cases over two or more weeks. If necessary, the learning groups would discuss cases with their tutors and would review the relevant theoretical background in medical genetics. Role-playing counselors and counselees in the classrooms discussed the genetic problems and solutions. Classmates participated in the discussion and question period. Tutors offered comments only at the close of the session. The teaching process was accomplished by students (*yellow*) or/and tutors (*green*)

A case was randomly assigned to a learning group (Fig. 2). The learning groups prepared the cases over a period of two or more weeks. If necessary, the leaning groups would discuss the cases with their tutors and would review the basic theories of medical genetics before their performance.

In every classroom, a tutor was responsible for 6–8 groups during about 2 h. During this process, the tutor did not lecture, but instead helped the students to evaluate whether their simulations were reasonable. The tutor encouraged all students to participate in the role-playing and discussion, and provided guidance to keep them on topic, without directly interrupting the performances. Before the tutor offered feedback as a lesson summary in the final 5–10 min of the class, undergraduates devoted over 80 % of class hours to participate the student-centered progress. One group played their roles in the front of the classroom, while the others watched their performance and participated in the discussion. Students were neither required to remain in their seats nor raise their hands before speaking. Role-playing counselors and counselees sometimes used self-prepared props or body language to add authenticity to their roles. During the role-playing section, counselors and counselees in the classrooms were required to play their roles including the following topics in each of the scenarios:

i) The causes of the disease,
ii) The type of inheritance,
iii) The risk to the offspring,
iv) The suggestions to the counselees and their families,
v) The best strategies for prevention and therapy,
vi) The methods used to detect specific genetic anomalies,
vii) Other related issues.

After the role-playing by every group, the other observation learners were permitted to question counselors and counselees or discuss the case and performance with them. A self-evaluation questionnaire was collected and a post-training instructional test was administered to the selected participants for the purpose of the research.

### Investigation of attitudes

To investigate attitudes concerning this program, a brief questionnaire was randomly administered to 324 students from 2009 to 2011 immediately after they had finished their training. Students answered the questions with *yes* or *no*. The validly completed questionnaires were recorded.

### Pre- and post-training instructional tests

To obtain the information concerning teaching efficacy, 200 participants from 2009 to 2011 were randomly subjected to pre- and post-training instructional tests. Both tests assessed understanding of the same 42 key points of genetic counseling. Tutors evaluated each student’s responses and rated them using 4 ranks (0 = unknown, 1 = poor, 2 = qualified, 3 = excellent). The average rank for each key point was compared between pre- and post-tests.

### Comparison of scores obtained in final examinations

To further measure the impact attributable to role-playing, the scores in final examinations of 578 program participants from 2009 to 2011, and those of 614 non-participating students from 2006 to 2008, were analyzed. The sample pools were matched with respect to age, sex ratio, place of origin, and entrance examination scores. The final examination for Medical Genetics was a standard exam graded using a hundred-mark system, was not significantly different between years, and included multiple choice questions, term explanations, short answer, and essay questions. Exam papers were critically evaluated according to standardized answers.

### Data Analysis

Data were analyzed using SPSS (version 12.0 for Windows) from IBM. The data were summarized by general statistical description in Investigation of attitudes, then compared using a paired *t* test in Pre- and post-training instructional tests, and by One-way ANOVA and two-tailed *t* test in Comparison of scores obtained in final examinations. Differences were deemed statistically significant when p < 0.05.

### Results

#### Medical undergraduates had positive attitudes to the role-playing component of the genetic counseling training program

**Fig. 3:**
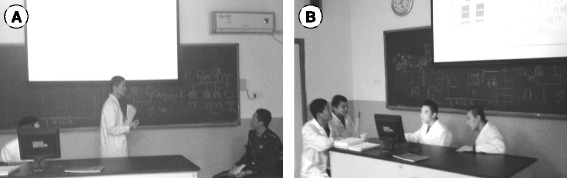
Face-to-face role-playing in the genetic counseling training program at TMMU. The program was student-centered. All learning groups had over 80 % of class hours to play their roles and discuss the cases in the role-playing section. During role-playing, counselors and counselees were permitted to use self-prepared props or body language to enhance role authenticity. The undergraduates shown in the figure were playing the roles of counselors and counselees in case scenarios involving hemophilia A (**a**) and β-thalassemia (**b**)

The genetic counseling training program at TMMU offers many contrasts with traditional pedagogy in China. The atmosphere in the classroom is open and free. Students take the “leading roles” in the classrooms while their tutors act in the capacity of the “audience” (Fig. 3), who fully experienced a student-centered training program. These might change the students’ attitudes to our training program.

Participants’ attitudes to this program have been closely monitored since it began in 2009. As shown in Table 2, both the training program and the instructional strategy of role-playing were positively received by over 97 % of the respondents. Over 75 % thought that role-playing was helpful in mastering medical genetics and genetic counseling, and in improving communication skills; over 70 % believed that the program would be useful in future professional careers. The data clearly indicated that students had positive attitudes toward the role-playing component of the genetic counseling training program at TMMU.

**Table 2:** Post-Training Questionnaire to assess Undergraduate Attitudes to the Genetic Counseling Training Program at TMMU, % Who Answered Yes (*N* = 324)

I ……	% yes
was willing to participate in this program more than in the traditional lectures	98.5
was glad to accept the instructional strategy of role-playing	98.5
felt that it was a pleasant experience to play the roles	97.2
agreed that role-playing was a good teaching method to help me understand what genetic counseling is and which processes and factors are involved	80.9
believed that role-playing could help me to master the knowledge of medical genetics by self-study	75.9
believed that role-playing could improve my communication skills with counselees or patients	79.0
believed that this program could help me in my future professional career	70.4

#### Genetic counseling training was effectively accomplished by role-playing

To assess program efficacy, tests were administered randomly to 200 participants from 2009 to 2011 before and after training. Analysis showed that the average levels of understanding exhibited by students for 40 key elements of genetic counseling had increased significantly after training (Table 3, Additional file 1 and 2). Only two elements showed no apparent change, and both were associated with materials that students had mastered through other courses. After training, most students performed at an excellent level, indicating that role-playing was an effective instructional strategy in genetic counseling training.

**Table 3:** Impact of role-playing on mastery of key concepts in genetic counseling (*N* = 200)

No.	Key concepts of genetic counseling	Average rank in		Change	(95 % CI)	Paired *t* test *P* value
		Pre-test	Post-test			
1	Congenital disease	2.05 ± 0.605	2.90 ± 0.308	-0.85	(-1.12 -0.58)	<0.0001
2	Familial disease	2.10 ± 0.6411	2.95 ± 0.224	-0.85	(-1.12 -0.58)	< 0.0001
3	Genetic disease	2.15 ± 0.587	3.00 ± 0.000	-0.85	(-1.12 -0.58)	< 0.0001
4	Genetic maker	0.30 ± 0.470	2.85 ± 0.366	-2.55	(-2.83 -2.27)	< 0.0001
5	Epigenetic	0.70 ± 0.979	2.85 ± 0.366	-2.15	(-2.68 -1.62)	< 0.0001
6	Mitosis	2.85 ± 0.366	3.00 ± 0.000	-0.15	(-0.32 0.02)	0.0828 (NS)
7	Meiosis	2.85 ± 0.366	3.00 ± 0.000	-0.15	(-0.32 0.02)	0.0828 (NS)
8	Dynamic mutation	0.50 ± 0.827	2.75 ± 0.444	-2.25	(-2.62 -1.88)	< 0.0001
9	Euchromatin	2.25 ± 0.910	2.95 ± 0.224	-0.70	(-1.13 -0.27)	0.0031
10	Heterochromatin	1.85 ± 0.933	2.85 ± 0.366	-1.00	(-1.48 -0.52)	0.0003
11	Sex chromatin	2.35 ± 0.875	2.95 ± 0.224	-0.60	(-0.95 -0.25)	0.0021
12	Karyotype	0.40 ± 0.503	2.85 ± 0.366	-2.45	(-2.73 -2.17)	< 0.0001
13	Pedigree	0.50 ± 0.688	2.95 ± 0.224	-2.45	(-2.77 -2.13)	< 0.0001
14	Trisomy 21 syndrome	2.10 ± 0.718	3.00 ± 0.000	-0.90	(-1.24 -0.56)	< 0.0001
15	Trisomy 18 syndrome	0.30 ± 0.571	2.65 ± 0.489	-2.35	(-2.73 -1.97)	< 0.0001
16	Trisomy 13 syndrome	0.25 ± 0.444	2.60 ± 0.503	-2.35	(-2.66 -2.04)	< 0.0001
17	Turner syndrome	0.80 ± 0.894	2.95 ± 0.224	-2.15	(-2.56 -1.74)	< 0.0001
18	Klinefelter syndrome	0.05 ± 0.224	2.75 ± 0.444	-2.70	(-2.97 -2.43)	< 0.0001
19	Huntington chorea	1.65 ± 0.875	2.60 ± 0.754	-0.95	(-1.53 -0.37)	0.0027
20	Albinism type I	2.00 ± 0.562	2.75 ± 0.444	-0.75	(-1.05 -0.45)	< 0.0001
21	Vitamin D resistant rickets	1.95 ± 0.605	2.55 ± 0.510	-0.60	(-0.98 -0.22)	0.0040
22	Duchenne muscular dystrophy	0.80 ± 0.696	2.20 ± 0.696	-1.40	(-1.75 -1.05)	< 0.0001
23	Heterogeneity	0.25 ± 0.444	2.90 ± 0.308	-2.65	(-2.88 -2.42)	< 0.0001
24	Genetic anticipation	0.15 ± 0.366	2.90 ± 0.308	-2.75	(-2.96 -2.54)	< 0.0001
25	Genetic imprinting	0.15 ± 0.366	2.85 ± 0.366	-2.70	(-2.97 -2.43)	< 0.0001
26	Delayed dominance	0.05 ± 0.224	3.00 ± 0.000	-2.95	(-3.05 -2.85)	< 0.0001
27	Irregular dominance	0.05 ± 0.224	2.90 ± 0.308	-2.85	(-3.02 -2.68)	< 0.0001
28	Incomplete dominance	0.75 ± 0.639	2.95 ± 0.224	-2.20	(-2.53 -1.87)	< 0.0001
29	Gene pleiotropy	0.50 ± 0.688	2.80 ± 0.523	-2.30	(-2.64 -1.96)	< 0.0001
30	Quantitative trait	0.05 ± 0.224	3.00 ± 0.000	-2.95	(-3.05 -2.85)	< 0.0001
31	Qualitative trait	0.05 ± 0.224	3.00 ± 0.000	-2.95	(-3.05 -2.85)	< 0.0001
32	Genetic susceptibility	0.20 ± 0.410	3.00 ± 0.000	-2.80	(-2.99 -2.61)	< 0.0001
33	Genetic liability	0.05 ± 0.224	3.00 ± 0.000	-2.95	(-3.05 -2.85)	< 0.0001
34	Coefficient of relationship	0.35 ± 0.489	3.00 ± 0.000	-2.65	(-2.88 -2.42)	< 0.0001
35	Inbreeding coefficient	0.35 ± 0.489	2.95 ± 0.224	-2.60	(-2.84 -2.36)	< 0.0001
36	Law of genetic equilibrium	0.40 ± 0.598	3.00 ± 0.000	-2.60	(-2.88 -2.32)	< 0.0001
37	Mitochondrial disease	1.25 ± 0.851	2.60 ± 0.503	-1.35	(-1.73 -0.97)	< 0.0001
38	Genetic hypothesis of tumorigenesis	0.60 ± 0.598	2.85 ± 0.366	-2.25	(-2.55 -1.95)	< 0.0001
39	Gene therapy	1.15 ± 0.489	2.80 ± 0.410	-1.65	(-2.00 -1.30)	< 0.0001
40	Genetic diagnosis	1.25 ± 0.550	2.85 ± 0.366	-1.60	(-1.88 -1.32)	< 0.0001
41	Genetic counseling	1.00 ± 0.725	2.80 ± 0.410	-1.80	(-2.19 -1.41)	< 0.0001
42	Genetic screening	0.80 ± 0.768	2.55 ± 0.510	-1.75	(-2.20 -1.30)	< 0.0001

*NS* Not Significant (*P* > 0.05)

#### Genetic counseling training by role-playing broadly affects education in Medical Genetics

**Fig. 4:**
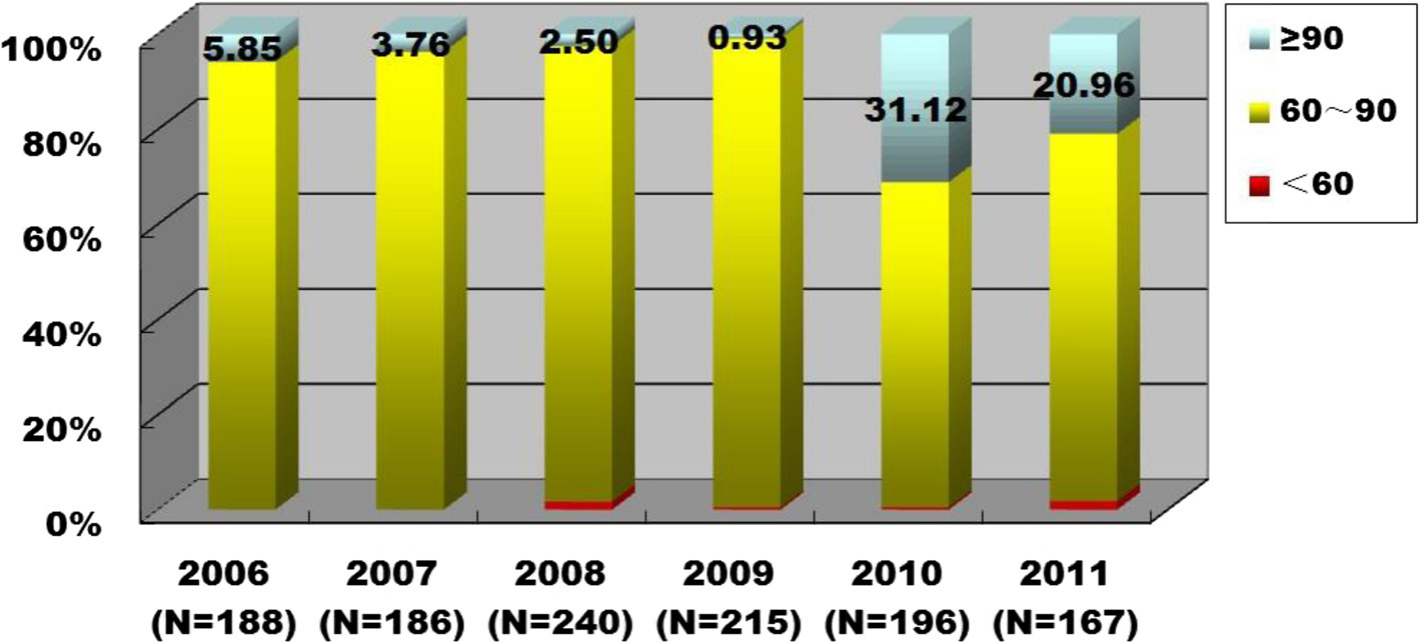
Analysis of student scores in final examinations for Medical Genetics from 2006 to 2011. Scores obtained in final examinations of 578 program participants (years 2009 to 2011), and those of 614 non-participating students (i.e., prior to the launch of the program, years 2006 to 2008), are compared. The percentages of student scores in three ranges (based on a one hundred-mark system) are shown as ≥90 (*blue*), 60 to 90 (*yellow*), and <60 (*red*)

The introduction of role-playing into genetic counseling training might affect other program components such as teaching and learning methods, student interest in genetics, motivation for self-study and active learning. Although these changes would be difficult to measure in isolation, collectively their effects could improve student scores in the Medical Genetics final examinations. To examine this hypothesis, final examination scores for 578 participants from 2009 to 2011, and scores for 614 non-participants from 2006 to 2008, were compared (Additional file 3). As shown in Table 4, the scores were significantly elevated among students who had participated in the training program. The percentage of student scores above 90 significantly increased after the role-playing training program had been initiated (Fig. 4). The data indicate that the genetic counseling training program by role-playing is an effective instructional strategy to improve the quality of medical genetics instruction for medical undergraduates.

**Table 4:** Comparison of student scores in final examinations for Medical Genetics from 2006 to 2011

Grades (N)	Mean ± SD	Multiple Comparison Test Significant? /Change (95 % CI)					Combining participants and non-participants			
		2010	2009	2008	2007	2006	Mean ± SD	Change	(95 % CI)	*t* test *P* value
2011 (*N* = 167)	82.2 ± 9.69	Yes/-3.58 (-5.92 -1.25)	Yes/4.97 (2.68 7.25)	Yes/5.93 (3.69 8.16)	Yes/7.32 (4.95 9.68)	No/2.15 (-0.208 4.51)	81.6 ± 8.44 (*N* = 578)	4.6	(3.62 5.50)	< 0.0001
2010 (*N* = 196)	85.8 ± 6.89	–	Yes/8.55 (6.36 10.7)	Yes/9.51 (7.38 11.6)	Yes/10.9 (8.63 13.2)	Yes/5.73 (3.47 8.00)				
2009 (*N* = 215)	77.3 ± 6.36		–	No/0.960 (-1.12 3.04)	Yes/2.35 (0.13 4.57)	Yes/-2.82 (-5.03 -0.605)				
2008 (*N* = 240)	76.3 ± 7.94			–	No/1.39 (-0.78 3.56)	Yes/-3.78 (-5.94 -1.62)	77.0 ± 8.03 (*N* = 614)			
2007 (*N* = 186)	74.9 ± 8.46				–	Yes/-5.17 (-7.46 -2.88)				
2006 (*N* = 188)	80.1 ± 6.76					–

## Discussion

### Educational reform and the change from traditional teaching to a student-centered program

Most departments of medical genetics at Chinese universities were established after 1978 [23]. Courses in medical genetics formally began in 1979 [23] and utilized lectures as the predominant teaching method. However, it was becoming increasingly clear at the time that new educational paradigms might result in substantially improved, compared to those obtained using traditional teacher-centered education or passive learning models [24–26]. In order to shift from traditional teaching modes to a student-centered program, at least two reforms were necessary. The first essential reform was to provide more time to students for independent study and to establish and reinforce the habit of self-study [27]. The second was restructuring the system of teacher enrollment and training because teachers remained irreplaceable in this program and they still shouldered important responsibilities [28].

### An opportunity for medical undergraduates to elevate communication skills, empathy and interest in genetics

To provide genetic counseling as a medical service, qualified counselors need both professional knowledge and excellent communication skills [8, 29], but medical undergraduates in the past had no opportunities to learn about the practice of genetics counseling, or to practice the skills required for effective communication. Role-playing has been defined as “an experimental learning technique with learners acting out roles in case scenarios to provide targeted practice and feedback to train skills”. It has been proven to efficiently develop communication skills in many disciplines and with learners of different backgrounds [30]. In the TMMU training program, role-playing students acted as counselors and used their professional knowledge to answer counselees’ questions and address their concerns. The role-playing counselees sometimes intentionally adopted characters with lower educational levels, forcing their partners to use simple words to explain professional terms and complicated theories. The students realized that professional communication skills made it possible to assist counselees by being emotionally supportive and by providing accurate information about genetic disease [8, 29].

Medical theories were over-emphasized in past decades while communication skills between doctors and patients were neglected [31]. This bias is partially responsible for conflicts between doctors and patients in China [32]. By playing the role of counselees, their caregivers, or their relatives, the undergraduates came to understand the physiological and psychic pain experienced by patients and the financial burden placed on their families [33]. After the course, some students commented that they would not be “medical robots” but would be more humane doctors in the future. Therefore, our project confirmed that role-playing allowed students to place themselves in scenarios that they had not previously experienced, allowing them to improve their empathetic abilities and better understand the motivations of others [6].

Most students had positive attitudes toward the training (Table 2). Furthermore, our program increased student interest in genetics, an effect also reported by Takemura and Kurabayashi in another role-playing exercise [34]. Their interest was induced not only by the fun of role-playing, but also by the opportunity to apply their knowledge to clinical problems, which clearly had practical value. To play the roles, students were required to connect isolated theories and apply comprehensive knowledge, and in some cases to develop new skills to interpret medical content on Wikipedia as well as to search PubMed, similar to what was described by Singh [35] in the context of physiology seminars. This had significant positive effects on learning (Tables 3 and 4 & Fig. 4). Efforts were made to improve student interest because Kumaravel [36] suggested that this could change attitudes toward genetic counseling and ultimately affect career choices.

### Limitations of the genetic counseling training program at TMMU

The first limitation was the number of class hours available for role-playing. The basic components of genetic counseling are informational and educational, which make training a time-consuming process [37]. At TMMU, more than 200 undergraduates typically participated in the program at one time. They are divided into eight classes of 24–26 students and play their roles during the 2-h face-to-face session. The tutors had little time to swap student roles between counselors and counselees, and consequently were uncertain whether every student was fully exercised. The time constraint also limited the types and numbers of case scenarios that could be explored. Some important cases, involving chromosomal and complex diseases, could not be included in the program. These limitations reduced training effectiveness.

The second limitation was the absence of an assessment for student role-playing performance. Students were judged exclusively by examination of their theoretical knowledge. Because their performance in the role-playing component of the genetic counseling program was ungraded, some students were reluctant to participate in the training.

The third limitaiton was the remained gap between the genetic counseling training and medical practice. Although the trained undergraduates had a solid knowledge basis, there were still other aspects of counseling that were not accounted for in this study, such as religion and tradition, legal issues, medical insurance, interpretation of big data, etc. The real counseling between actual patients or counselees and genetic counselors could not be completely simulated by the present role-playing training.

## Conclusions

Beginning in 2009, TMMU has offered a student-centered genetic counseling training program that enables third-year medical students to apply theoretical knowledge to clinical problems. The training program, using the instructional strategy of role-playing, was accepted by most students and had significant positive effects on their mastery of key components in genetic counseling. We conclude that this program offers a feasible and effective teaching method for training genetic counselors in mainland China as well as in other developing countries.

## Additional files

Additional file 1: Students’ ranks in pre-training instructional tests on 42 key elements of genetic counseling. (XLS 77 kb) Additional file 2: Students’ ranks in post-training instructional tests on 42 key elements of genetic counseling. (XLS 75 kb) Additional file 3: Scores in final examination for Medical Genetics from 2006 to 2011. (XLS 34 kb)

## Abbreviations

TMMU: Third Military Medical University
